# Supplementary material for: Are carbon emissions trading and green financial instruments synergistic? -Comprehensive quantitative research based on content analysis
Source: PLoS One. 2024 Mar 7;19(3):e0298601. doi: 10.1371/journal.pone.0298601 (PMC10919601; doi:10.1371/journal.pone.0298601)
Supplement: S1 Data — (ZIP) [file pone.0298601.s001.zip › Data/地级市Z_F工作报告.zip!/║■─╧/┬a╡╫╩╨/└┤╫╘╣1⁄2╓┌║┼í╛┬φ┐╦╩2╛▌═°í┐_2009.docx]

各位代表：

现在，我代表市人民政府，向大会作政府工作报告，请予审查，并请各位政协委员和其他列席人员提出意见。

一、过去五年工作回顾

2004年至2008年，是我市历史上发展速度最快、城乡变化最大、群众受惠最多的五年。五年来，在市委的坚强领导下，在市人大、市政协的监督支持下，全市上下深入贯彻落实科学发展观，坚持“与时俱进、后发赶超”，抢抓机遇，真抓实干，战胜历史罕见的低温雨雪冰冻等多种灾害，灵活运用宏观调控政策，积极应对国际金融危机，较好地完成了市二届人大一次会议确定的各项目标任务，开创了我市经济社会发展的崭新局面。

（一）综合实力实现新跨越

2008年，实现生产总值550亿元（预计数，下同），是2003年的2.6倍，年均增长12.6%，总量在全省由第12位上升到第10位。三次产业比为20.9:48.4:30.7，第二产业比重五年提高7.8个百分点。2008年完成财政总收入40.03亿元，是2003年的2.97倍，

年均增长24.3%，完成一般预算收入22.3亿元，是2003年的2.44倍，年均增长19.6%，均由全省第11位上升到第8位。2008年完成全社会固定资产投资184亿元，为2003年的2.37倍，年均增长18.8%。五年利用内资236.3亿元、引进外资5.15亿美元，年均分别增长49.5%、18%。2008年完成进出口总额10.32亿美元，为2003年的9倍，跃居全省第3位。2008年全市金融机构各项存款余额485亿元、贷款余额241亿元，分别为2003年的2.32倍、2.2倍，年均分别增长18.3%、17.3%。

新型工业化加快推进，597家国有企业改革基本完成，9.48万名职工置换了身份，以湖南宜化、湖南海螺为代表的改制重组企业重现活力。非公经济快速发展，增加值占GDP的比重上升到41%。大力推进园区建设，以市经济开发区为代表的各类园区正成为经济发展新的增长点。农业产业化成效明显，2008年农产品加工企业达到853家，其中市级以上龙头企业92家。科技创新能力明显增强，五年获得省级以上科技成果179项，申请专利2152件，2007年被列为全国知识产权试点城市，科技进步综合水平居全省第5位，科技对经济增长的贡献率达到46.9%。

加快推进对外开放与文化交流，城市软实力与知名度显著提升。与美国拉姆西市、伊甸园市结为友好城市，成功引进了沃尔玛、香港华润、三一重工、湖北宜化、安徽海螺和台湾国产实业等战略投资者，“坚韧、勤奋、尚德、自强”的娄底精神得到彰显，戏剧《村官是个堂客们》获得全国映山红金奖，紫鹊界梯田列入世界自然与文化双遗产预备名录，曾国藩故居列为国家重点文物保护单位。

（二）城乡面貌呈现新变化

五年完成基础设施投资226亿元，2008年完成65亿元，为2003年的6.5倍。交通状况明显改善，改建干线公路183公里，公路总里程达到10606公里，娄新高速、二广高速娄底段开工建设，县乡公路基本实现油路或水泥路，98%的行政村通了公路。

娄底中心城区“北扩南移”加速推进，湘中大道、吉星路南段、第一生活污水处理厂、生活垃圾填埋场、垃圾中转站、春园商业步行街、五江建材城等先后建成，城区道路骨架扩展到60平方公里，建成区面积达到40多平方公里，城市人口增加到36万。县市城区和小城镇建设明显加快，全市城市化率达到36.1%，五年年均提高1.58个百分点。

新农村建设加快推进，建立了领导挂点、部门共建、企业帮扶示范村建设制度，78个新农村建设示范村列入全省“千村示范”工程。农村道路、水利、电网、通信建设得到加强，五年改造农村公路6466公里，完成6座中型水库除险加固，解决21.34万人的饮水困难和29.4万人的饮水安全问题。

生态环境保护明显加强，连续五年实现耕地占补动态平衡，项目用地得到优先保障，整顿和规范矿产资源开发秩序取得明显成效，采煤采矿沉陷区加快治理，万元GDP能耗年均下降5%，全市森林覆盖率达到41.8%。在成功创建省级园林城市、卫生城市、文明城市和全国绿化模范城市后，先后入选全国十佳宜居城市、中国最佳生态旅游城市，青春娄底展现新姿。

（三）民生改善取得新成效

2008年城镇居民人均可支配收入12200元、农民人均纯收入3250元，分别比2003年增加5658元、1372元，年均分别增长13.3%、11.6%。2008年实现社会消费品零售总额150亿元，年均增长9%。五年新建廉租房19万平方米、经济适用房28万平方米，城市人均居住面积由25.7平方米增加到31.1平方米。

全面免除义务教育阶段学生学杂费，五年新建扩建校舍72.5万平方米，改造学校危房74万平方米，“普九”成果不断巩固，高考上线率连续五年全省第一，娄底师专成功升本，建成娄底职业技术学院、潇湘职业技术学院，基础教育、职业教育、成人教育、高等教育协调发展。

加强劳动力就业培训，五年新增城镇就业15.76万人、农村劳动力转移31.85万人，养老、失业、医疗、工伤和生育保险覆盖面不断扩大。建立城乡医疗救助制度，新改扩建乡镇卫生院93所、城市社区卫生服务中心11所、疾控中心和传染病区10所，推行新型农村合作医疗制度，启动城镇居民基本医疗保险试点，城乡医疗服务水平不断提高。

加快扶贫开发，救助弱势群体，五年落实库区移民资金9.45亿元，31.56万库区移民生活水平稳步提高，9.96万农村贫困人口稳定脱贫。城市低保实现动态管理下的应保尽保，农村低保全面启动，新建改建敬老院和五保之家114所。解决农村广播电视盲区群众听广播、看电视14.99万人。

人口与计划生育工作进入全省先进行列，人口自然增长率稳定在7.6‰以下。切实抓好安全生产，建立完善安全生产长效机制，安全形势持续好转，五年来安全生产事故逐年减少，死亡总人数下降58%。加强信访维稳和社会治安综合治理，平安娄底创建活动全面推进，居民安全感不断提升，社会和谐稳定。

（四）依法行政得到新加强

加快转变政府职能，深入贯彻行政许可法，大力推进行政管理体制改革，逐步建立和完善公共财政、公共服务和应急管理体系。自觉接受人大及其常委会的法律监督、工作监督，政协的民主监督和社会舆论监督，五年共办理市人大代表建议与议案714件、市政协委员提案847件，2008年办结率、面商率和满意率均达到百分之百。

加强公务员队伍建设，转变公务员作风，为民服务意识不断增强。严格执行党风廉政建设责任制，坚持不懈地加强反腐倡廉建设，集中治理商业贿赂，依法查处违法违纪案件，政府公信力不断提升。科学应对自然灾害、物价上涨、金融危机等各种严峻考验，政府驾驭复杂局面、市场经济和领导发展的能力不断增强。

军队和国防后备力量建设稳步推进，在全市经济社会发展特别是抗灾救灾和维护社会稳定中作出了重要贡献。监察、人事、审计、统计、物价、民族、宗教、对台、外事侨务、贸促、质监、食品药品监督、地方志、档案、保密、气象、地震、人防、仲裁和工会、共青团、妇女、儿童、老龄、残联、红十字会等各项工作均取得了新的进步。

回顾五年发展历程，我们深切体会到，这些成绩的取得，得益于市委的坚强领导，得益于市人大、市政协的监督支持，得益于各级各部门和社会各界的团结合作，得益于全市人民的共同努力。在此，我代表市人民政府，向全市人民，向各位人大代表、政协委员，向各民主党派、工商联、无党派人士、各人民团体和社会各界人士，向驻娄人民解放军、武警部队官兵、政法干警，向各位离退休老领导、老同志，向所有关心支持娄底建设和发展的朋友们，表示崇高的敬意和衷心的感谢！

各位代表，五年来，我们始终坚持解放思想，矢志加快发展，在实践中积累了许多宝贵经验，主要是：

一是必须积极抢抓机遇，加快赶超步伐。五年来，我们始终把抢抓机遇作为赶超的首要环节，立足市情，顺应形势，抢抓机遇，努力用好用活国家政策，争取实施了一批大项目、好项目，实现了全市经济社会的大发展。

二是必须推进改革开放，创新体制机制。五年来，我们始终把改革创新作为攻坚克难的重要法宝，以壮士断腕的决心推进国企改革，狠抓农村、财税、教育、科技、卫生、社会保障等各项改革，不断扩大开放，为全市经济社会发展增添了新的活力。

三是必须坚持工业主导，强化“一化三基”。五年来，我们始终坚持工业主导，着力基础产业，主攻基础设施，加强基础工作，加快推进新型城市化和农业现代化，进一步夯实了发展基础，增强了发展后劲。

四是必须突出改善民生，凝聚发展力量。五年来，我们始终坚持以人为本，把实现好、维护好、发展好群众利益作为工作的出发点和落脚点，全面落实惠民政策，切实为民办好实事，使政府工作赢得了广大群众的拥护和支持。

五是必须加快职能转变，优化发展环境。五年来，我们始终坚持把转变职能、改进作风作为加快发展的重要保障，持之以恒地推进政府职能转变，加强机关作风建设，提高政务服务水平，优化投资创业环境，推动了全市经济社会又好又快发展。

这些宝贵的经验，我们在今后的工作中要继续坚持和发展。

在肯定成绩的同时，我们也清醒地看到，我市经济社会发展中还存在不少矛盾和问题，主要是：思想解放还不够，发展氛围还不够浓；经济总量还不大，产业结构还不优，发展速度还不够快；基础设施相对滞后，投资规模相对较小，生态环境相对脆弱；政府职能转变还不到位，干部作风还不够实；社会矛盾还不少，民生改善的任务还很重。所有这些，都需要我们高度重视，并在今后的工作中切实加以改进和解决。

二、今后五年工作指导思想和目标任务

当前，国际金融危机的影响继续加深，全球经济增长明显减速，世界经济环境中不确定因素和突发性风险前所未有，我国经济下行压力明显加大，我市经济发展的外部环境可能更加严峻。但我们更要看到，今后五年，是我市科学发展、加速赶超的关键时期。放眼全球，各国为应对金融危机促进发展均在采取积极举措，国际产业、资本、技术转移与合作仍在加快。纵观全国，在国家保增长等一系列政策的推动下，我国经济增长仍将保持在较高区间，经济社会发展的基本面没有改变。环看省内，随着“一化三基”战略的深入实施和长株潭“两型”社会建设的加快推进，全省各市州竞相发展的形势咄咄逼人。审视自身，我市经过近几年的赶超发展，环境明显改善，基础明显夯实，实力明显增强，已经站在新的历史起点上。所有这些，都为我市加快发展提供了千载难逢的历史机遇。

面对挑战和机遇，我们必须坚定信心，迎难而上，立足全市人民奋力拼搏形成的发展基础，发扬全市人民坚韧勤奋的优良传统，珍惜全市上下奋发向上的大好局面，不负全市人民加快富民强市的迫切愿望，全面提升发展理念，深入推进改革创新，不断开创经济社会发展的新局面。

今后五年政府工作的指导思想是：全面贯彻党的十七大、十七届三中全会精神，以邓小平理论和“三个代表”重要思想为指导，深入学习实践科学发展观，按照省委省政府“一化三基”战略和市委三届四次全会精神要求，始终突出“科学发展、加速赶超”主题，大力实施“工业主导、基础先行、项目带动、协调发展”战略，继续解放思想、深化改革、扩大开放、务实创新，为建设富裕文明和谐娄底而努力奋斗。

经济社会发展的奋斗目标是：生产总值年均增长11.5%以上，到2013年，达到1100亿元以上；人均生产总值达到23500元以上；财政总收入年均增长14%以上，到2013年，突破70亿元，其中一般预算收入年均增长13%以上，到2013年，突破40亿元；全社会固定资产投资年均增长18%以上；社会消费品零售总额年均增长15%以上；城镇居民人均可支配收入年均增长7%以上，农民人均纯收入年均增长8%以上；人口自然增长率控制在7.6‰以下。初步建成新型能源原材料基地、特色装备与先进制造业基地、文化与生态旅游休闲基地、区域性商贸与物流中心、区域性交通枢纽（三基地一中心一枢纽）。

实现今后五年发展目标，要重点做好四个方面的工作：

（一）坚持工业主导，加快推进“三化”进程

突出工业在三次产业中的主导地位，把新型工业化作为富民强市的第一推动力，引领带动新型城市化和农业现代化。

大力促进信息化与工业化融合，狠抓传统产业优化升级，着力培育新兴产业，整合提升产业园区，加快推进自主创新，做大做强产业集群。抓好冶金、能源、建材、化工、机械五大传统产业的优化升级，整合提升特种陶瓷、矿山机电、农用机电三大特色产业；加快培育新材料、新能源、先进制造、电子信息、生物医药等高新技术产业，鼓励和支持骨干企业开展自主研发和科技创新；积极实施“质量兴企、品牌强市”战略，力争在创中国名牌产品和国家驰名商标上有新突破。重点做大精品钢材及薄板深加工产业集群，集中打造装备制造、煤炭深加工、建材、化工、有色冶炼、食品深加工等百亿产业。到2013年，实现工业增加值500亿元，年均增长14%以上，力争建成1－2个产值达100亿元的产业园区。

坚持高起点规划、高标准建设、高效能管理，加强城市规划，提高规划水平，抓好规划落实，维护规划的权威性、严肃性和可持续性。强化城市功能规划与产业规划的衔接，积极推进城市产业发展，以产业支撑城市功能，集聚城市人气，有效提升城市综合竞争力。重点推进中心城区和“娄底—双峰”、“娄底—涟源—冷水江—新化”城镇带建设，提升中心城区的功能，加快县市城区扩容提质，建设好15－20个重点中心镇，努力打造“3+5”城市群中独具特色的宜居宜业新城，使青春娄底更显活力。到2013年，力争全市城市人口达到202万以上，城市化率达到46.8%以上。围绕创建中国优秀旅游城市、国家卫生城市、国家园林城市目标，加强城市环境特别是软环境建设，推进城市精细化管理，改善市民居住环境，不断提高城市文明水平。

全面落实十七届三中全会精神，深化农村综合改革，加快推进新农村建设，大力发展现代农业，完善农村服务体系，促进农民持续增收。按照“优质、高效、安全”要求，壮大生猪和草食动物、优质稻、中药材、南竹、果蔬五大优势产业，大力发展油茶产业。严格耕地保护，落实惠农政策，加强双峰、涟源、新化等全国粮食生产基地县（市）建设，确保粮食稳产增产。力争五年内培育2－3家全国农业产业化龙头企业、2－3家年产值过10亿元的农产品加工企业，确保农业增加值年均增长4%以上。加强山洪地质灾害防御，提高自然灾害监测预警能力。落实农机购置补贴，提高农业机械化水平。加强农民培训，提高农民素质。继续实施新农村建设“千村示范”工程和农村清洁工程，改善村容村貌。

（二）坚持基础先行，加快优化发展环境

围绕“三基地一中心一枢纽”目标，加快基础设施建设，努力形成大交通、大水利、大电力、大通信体系，不断夯实科学发展、加速赶超平台。

加强交通路网建设，加快沪昆高速铁路娄底段建设、娄邵铁路既有线改造和冷水江火车东站扩建，建成娄新高速、二广高速娄底段，加快建设新怀高速，争取娄衡、娄长、益娄衡高速、冷水江资江二大桥进入省里规划并逐步开工建设，完成国道207和省道209、210、217、225、312、314等干线公路改造，推进通乡、通村、通畅工程建设，到2013年，新增高速公路249公里，公路总里程突破11800公里。

加强水利设施建设，着力抓好农村安全饮水、中型灌区续建配套、农村小型水利设施和城镇防洪工程建设，全面完成全市现有农村安全饮水和中小型病险水库治理任务，提高防洪抗旱减灾能力。加快白马灌区续建配套和新化梅山大灌区建设，加强资水、涟水、孙水综合治理，抓好中心城区和各县市城区饮用水源建设。

加强城乡电网、通信和广播电视建设，全面提升城乡承载力。五年完成电网建设投资27亿元，新建或扩建娄西50万伏变电站、新化西等9座22万伏变电站、琅塘等23座11万伏变电站，继续加快城网、农网建设与改造。实施有线电视村村通工程，五年内实现所有乡镇与县市城区联网。

（三）坚持项目带动，加快扩大投资和消费

把项目建设作为科学发展、加速赶超的第一抓手，抢抓机遇，多上项目，扩大投资和消费，增强经济发展后劲。

着力谋划一批基础产业、基础设施、生态环保、公共服务和民生工程项目，加快金电煤矸石发电、新化电厂、安康—张家界—娄底—衡阳铁路、张家界—桂林铁路娄底段、浙商物流园等重大项目前期工作。切实抓好涟钢汽车板、冷钢300万吨钢配套、金电二期2×60万千瓦、湖南宜化“315”工程、三一液压油缸扩改、天然气管输工程等重大项目。到2013年实现千万吨钢、千万吨煤和千万吨水泥的目标，培育3个年产值过100亿元、4个年产值过50亿元、15个年产值过10亿元的骨干企业。

以项目为载体，加快承接产业转移和央企对接，扩大对外开放，促进开放型经济再上台阶。突出延伸钢材产业链、电源电池新材料、有色冶炼、装备制造、旅游休闲等产业招商。加强招商项目的跟踪与落实，提高项目的履约率、开工率和投资到位率。力争五年利用外资6亿美元以上，利用内资500亿元以上；进出口总额年均增长12%以上。

加快发展第三产业，进一步扩大城乡消费。加强旅游资源的保护与开发，壮大发展旅游支柱产业，到2013年，旅游总收入突破75亿元，年均增长30%以上。加快发展金融、信息、会展、中介、物流等现代生产性服务业，规划提升房地产、餐饮娱乐等生活性服务业，积极开发文化创意、服务外包等新兴产业。重点围绕钢铁、煤炭、建材等优势产业培育一批竞争力较强的专业物流市场。加快社区建设，着力完善社区服务设施、服务网络，拓展社区服务领域，形成广覆盖、多层次、社会化的社区服务体系。着力完善城乡市场网络建设，推进现代流通方式向农村延伸，培育和扩大消费热点。到2013年，实现社会消费品零售总额300亿元以上。

（四）坚持协调发展，加快推进社会建设

按照全面、协调、可持续要求，统筹推动城乡区域、经济社会、人与自然协调发展。

突出民生改善，健全社会保障体系。有效促进和扩大就业，积极培育创业主体，以创业带动就业，到2013年，城镇累计新增就业岗位18万个以上，新增农村劳动力转移就业25万人以上。加强社保资金的征缴和管理，逐步提高企业离退休人员基本养老金标准和城乡低保补助标准，实现城镇从业人员基本养老、医疗保险全覆盖，失业保险覆盖率提高到95%以上。完善住房保障体系，扩大住房公积金覆盖面，加快城镇保障性住房建设和农村安居工程建设，着力解决低收入群众住房困难。

协调发展社会事业，继续实施“科教兴市”战略，大力推进创新型娄底建设，加快“教育强市”、“人才强市”步伐。到2013年，学前三年毛入园率达到72%以上，义务教育完成率100%，高中阶段教育毛入学率90%以上；高新技术产值年均增长28%以上，到2013年突破260亿元。发展文化体育事业，积极做好省十二届运动会申办工作，建设市文化中心、市图书馆、市工人文化宫、市青少年活动中心、市妇女儿童活动中心、老街历史文化街。完成第二轮修志任务。完善公共卫生和医疗服务体系。扎实做好人口与计划生育工作，稳定低生育水平，改善人口结构，提高人口素质。大力发展妇女、儿童、残联、老龄等其他各项社会事业，促进经济社会全面进步。

认真落实市人大常委会关于加强环境保护工作的决定，切实把环境保护放到经济社会发展的先导位置，坚持源头控制、全程控制和总量控制，促进生态环境持续改善。加快推进节能减排，大力发展资源节约型、环境友好型产业，积极推进清洁生产，加大生态环境保护、战略水资源建设、污染源与矿山采空区治理力度，确保单位GDP能耗、化学需氧量、二氧化硫排放量逐年削减，达到省定目标。坚持土地集约节约利用，加强国土开发整理，统筹安排建设用地，确保耕地占补平衡。到2013年，全市森林覆盖率达到43.2%以上，城市生活垃圾无害化处理率90%以上，污水处理率80%以上。

牢固树立安全发展理念，坚持不懈地抓好安全生产，坚决防止重特大事故发生。切实加强食品、药品、农产品质量安全监管。完善信访工作机制，从源头上解决突出信访问题。加强和改善社会管理，积极推进城乡和谐社区和基层民主政治建设，打造平安娄底。加大对贫困县市和库区的扶持力度，支持县域经济发展，推动城乡一体化进程。

三、2009年工作重点

今年是新中国成立60周年，是我市撤地设市10周年，也是新一届政府的开局之年，做好全年工作，意义十分重大。综合各方面的情况分析，今年可能是我市经济发展最为艰难的一年。我们一定要按照市委经济工作会议精神，牢牢把握“保增长、扩内需、调结构、促就业、强基础”的总体要求，把保增长作为全年经济工作的首要任务，把扩内需作为保增长的根本途径，把调结构作为保增长的主攻方向，把促就业作为保增长的重要目标，把强基础作为保增长的有力支撑，进一步坚定目标，提振信心，因势利导，变压力为动力，化挑战为机遇，千方百计争取项目和资金，切实为民办好实事，全力推动全市经济社会又好又快发展。

今年我市经济社会发展的目标是：生产总值计划增长11%以上，工作目标12%以上，其中第一产业增加值增长4.5%以上，第二产业增加值增长13%以上，第三产业增加值增长11.5%以上；财政总收入增长14%以上，其中一般预算收入增长13%以上；全社会固定资产投资增长25%以上；进出口总额增长12%以上；城镇居民人均可支配收入增长7%以上，农民人均纯收入增长8%以上；人口自然增长率控制在7.2‰以内；城镇登记失业率控制在4.8%以内；亿元GDP生产安全事故死亡率控制在0.56以下；单位GDP能耗、主要污染物排放总量完成省定目标。

实现上述目标，今年要重点做好以下八个方面的工作：

（一）抢抓机遇加快工业结构调整

加大服务企业力度。进一步提高政府的服务能力和水平，帮助企业坚定发展信心，用好用足用活国家扩大规模与优化结构并举的投资政策、更加主动的贸易政策、更加优惠的税收政策，寻找新的发展机遇和投资空间。千方百计在资金、财税、用地等方面加大对重点企业的扶持力度，积极为企业排忧解难。加强与金融机构、担保公司的联系合作，设立中小企业担保基金，切实为企业特别是中小企业解决运转难、融资难等问题。加强政府对经济的预测、分析、调控与指导，为企业提供一流的服务，最大限度地帮助企业渡过难关、发展壮大。

加快产业优化升级。充分利用国家鼓励转变发展方式、加快产业产品结构调整的有利时机，加快煤炭产业向煤电、煤焦、煤化工转变，支持水泥等建材产业加快技术改造、提高效益，引导涟钢、冷钢、闪星锑业等企业适应市场变化，调整产品结构。加大技术创新力度，加快培育新兴产业，重点引进发展对提升我市产业结构有战略意义的科技密集、资本密集型产业，大力发展食品、服饰、医药等消费品产业。支持引导中小企业和非公有制企业加快发展。力争全年规模企业达到600家，实现工业增加值300亿元，增长13.4%以上，规模工业增加值增长15%以上，高新技术产业增加值占工业增加值的比重达到32%以上。

大力发展产业园区。完善管理体制，加强用地保障，拓宽融资渠道，加快基础设施建设，提高园区的产业承载力。加强园区规划与重点产业规划对接，完成产业园区控制性详细规划，促进优势产业向园区集聚。重点抓好市经济开发区和各县市区产业园区建设，力争市经济开发区成为省级高新区，娄星工业园区成为省级产业园区。确保全市工业园区完成固定资产投资增长30%以上，实现工业增加值增长28%以上。

（二）千方百计加快项目建设

创新项目工作机制。今年将作为项目建设年，快抓项目储备，巧抓项目争取，狠抓项目建设，严抓项目环境，在全市形成大抓项目、大干项目、大促项目的生动局面。建立项目目标管理制度，将项目谋划、储备、争取和项目建设任务，细化分解到县市区和市直责任单位，强化考核，加强督查，确保落实，为项目建设营造宽松环境。建立项目运行管理制度，设立专项资金，推动土地、资金、人才向项目倾斜。创新项目建设投融资机制，激活民间投资，扩大招商引资，形成多渠道、多元化的项目建设投入机制。

全力推进项目招商。坚持把招商引资作为加快项目建设的突破口来抓，紧盯产业转移动向，对接世界500强、央企和优质上市公司，争取引进一批战略投资者。办好湘中之光经贸博览会，加快引商入娄步伐。创新招商方式，突出龙头企业和产业链招商，开展项目招商、园区招商、专项招商和以商引商，提高招商实效。成立重大项目建设协调小组，营造和形成干部围着项目转、工作围着项目做的浓厚氛围，坚决打击阻碍项目建设、影响项目招商的违法违纪行为，切实优化招商引资和项目建设环境。力争全年实际利用外资9000万美元以上，引进内资72亿元以上，增长20%以上。

着力主攻重点项目。集中抓好全年在建的81个重大建设项目，完成投资153.3亿元。

工业及高新技术产业项目完成投资97.75亿元，重点抓好涟钢薄板、安石96万吨焦炉城市煤气改扩建工程、涟源国产实业水泥和科源煤矸石深加工等省“双百工程”项目；加快建设冷钢线材与炼焦、天宝紧固件、汇源焦化、双峰生物质能发电、涟源华润电力、金富源碱业节能技改、中源新材料、中兴液压油缸、金华汽车、湖南海螺二期、波月水泥等项目；积极促进红太阳电源电池、三星锻造、红宇耐磨、南方连铸高效结晶器铜管等高技术产业项目做大做强。

基础设施项目完成投资42.79亿元，重点抓好二广高速娄底段、娄新高速、新怀高速、娄邵铁路、冷水江火车东站、国省道干线公路改造等交通项目；加快白马灌区续建配套与节水改造，资水涟水孙水综合治理，新化、涟源、双峰城市防洪工程，农村安全饮水，小一型水库除险加固，新化22万伏鹅塘变电站，小型水电站等水电项目。加快娄衡高速、沪昆铁路客运专线开工建设的前期工作。

旅游业项目完成投资3.48亿元，重点抓好紫鹊界梯田—梅山龙宫、大熊山国家森林公园及蚩尤文化旅游区、曾国藩故居文化旅游综合开发、仙女寨生态旅游区、中华蚩尤始祖文化园、龙山华夏中药文化园、湄江岩溶地质公园、洪家山—双江水库生态旅游等项目。

社会事业项目完成投资4.99亿元，重点抓好市体育中心、市科技中心、市中心医院住院楼、市中医院综合楼、市有线数字电视、娄底技师学院、娄底卫校升专扩建、湖南人文科技学院基础设施等项目建设。

生态环保项目完成投资4.26亿元，重点抓好涟邵、冷水江、涟源采煤沉陷区治理、锡矿山有色金属棚户区改造、灾后重建、退耕还林等项目。

（三）促进农民持续增收

大力发展现代农业。确保粮食播种面积不低于445万亩、总产180万吨以上。做强“永丰辣酱”、“天源黑猪”、“天华黑牛”、“同星米业”等品牌，建设无公害水果基地5个、蔬菜基地4个、茶叶基地2个、中药材基地2个，低改新造油茶2.3万亩，做大做强农产品加工企业，加工优质米25万吨，力争农产品加工产值增长20%以上。实施无公害农产品和绿色食品计划，全年增加无公害农产品认证20个、绿色食品认证10个，大力发展农民专业合作组织，加大农机推广力度，不断提高农业机械化水平，增加农民参与农业产业化经营的收入。

完善农村服务体系。稳定农村土地承包制度长久不变，积极引导土地承包经营权依法有序流转，鼓励发展多种形式的适度规模经营。加快征地制度改革，健全对被征地农民的补偿和安置机制。有序推进集体林权制度、基层水管制度和农村金融制度改革，大力发展农村金融和农业保险，推进政策性农业保险试点工作。健全农村科技推广、动植物防疫、信息服务等社会化服务体系，农村科技合作组织入户率达到50%以上。落实农业各项财政补贴和涉农税费减免优惠政策，增加农民政策性收入。

加强农民就业培训。重点抓好农民实用技术培训、引导性培训和技能培训，培训农民26万人次以上，新增农民转移就业5万人。高度重视返乡农民工的就业问题，落实优惠政策，鼓励支持有技术、有资金、懂管理的返乡农民工自主创业。充分挖掘农村内部就业增收潜力，大力发展农村二、三产业和外派劳务，有效增加农民劳务收入。

推进新农村建设。坚持“政府引导、农民为主、三级共建”原则，完善工作机制，开展企业联村扶村活动，积极动员社会力量参与新农村建设。继续实施新农村建设“千村示范”工程，抓好村容村貌治理，全面启动村庄整治，在每个县市抓好一个以上清洁工程示范村建设。以提高农业综合生产能力为重点，坚持防汛抗旱并重，加强农田水利基本建设，完成水利投资3亿元。加大土地整理和中低产田改造力度，抓好新增粮食产能高标准粮田、优质粮食产业工程、退耕还林基本口粮田和沃土工程等重大项目建设。加强山洪地质等自然灾害防御，加快改善农村道路、电网、通信、饮水等基础条件。

（四）切实加强城市建设和管理

完善城市规划体系。完成中心城区城市总体规划修编和城市综合交通、城市绿地等专业规划，编制中心城区城市景观分区控制规划、潭邵连接线街道景观规划、娄星广场区域城市景观规划，完成涟水河、孙水河两岸风光带规划。加快完善县市城区综合交通、基础设施、公益性公共设施等专项规划，加强控制性详细规划和沿河等重点地段修建性详细规划，做好重点城镇、名胜风景区、历史文化名镇（村）规划编制工作。

加快城市基础建设。中心城区加快城市防洪堤和吉星路北端等主要干道建设，启动株山公园、甘桂路、高丰路、潭邵连接线改建四大项目，加快推进市经济开发区、城南新区、娄星经济园区配套道路建设。继续推进主次干道提质、电力通信设施改善和背街小巷改造。加快县市城区和小城镇建设，提高县城和建制镇的综合承载能力。以居民住得起房、买得起房为目标，落实国家优惠政策，鼓励合理住房消费，规范和促进房地产业持续健康发展。力争全市完成市政基础设施投资8亿元，其中中心城区完成5亿元，城市化率达到38.1%以上。

强化城市规范管理。加强城市依法有序管理，全面推行市县两级相对集中行政处罚权。加强城市环卫、园林绿化、客运公交、农贸市场、社区物业等规范管理，提高市民的主人翁意识。做好征地拆迁工作，维护好拆迁户的合法权益。严格规划执法，依法加大违规建筑的拆除力度，改善城市形象。加强村镇环境整治，改善人居环境，塑造村镇新面貌。

（五）大力发展第三产业

加快发展现代物流业。完善商业网点布局，积极引进大型流通企业，大力发展社区商业，着力培育物流配送、连锁经营、电子商务等新型业态，重点加快建设湘中综合物流园、湘中商贸物流配送中心和湘中粮食物流园，抓好会展中心和展览馆的筹划，提升服务业水平。抓好农产品批发大市场和农村流通设施建设与管理，继续实施“万村千乡”工程，落实好“家电下乡”等惠民政策，拉动农村消费。加快铁路口岸建设，改善基础条件，鼓励进出口，扩大对外贸易，完成进出口总额11.2亿美元。

积极发展金融服务业。深化投融资体制改革，健全中小企业融资担保体系，密切银政、银企关系，增强金融对地方发展的支持力度，推动优质公司加快上市步伐。有效防范金融风险，严厉打击非法集资行为，努力创建金融安全区，加快双峰村镇银行、娄星农村信用合作银行建设。鼓励支持发展保险、证券业。

壮大发展旅游和文化产业。认真落实市人大常委会关于加强旅游资源保护的决定，加大旅游资源的保护与开发力度，有效整合旅游资源，完善配套基础设施，全面提升旅游产品质量和市场竞争力，壮大旅游产业。重点加快紫鹊界梯田、梅山龙宫、曾国藩故居、波月洞、湄江和锡矿山工业旅游区、涟钢工业旅游区等旅游品牌建设，实现创建中国优秀旅游城市目标，力争全年旅游收入增长30%以上。大力加强文化建设，加快文化体制改革，建立覆盖全社会的公共文化体系，推进乡镇文化站、农家书屋、文化信息资源共享建设和农村公益电影放映工作，加强文物保护工作，加快双峰文化名人一条街建设，促进文化资源向经济资源转变。

（六）着力推进生态环境建设

大力推进节能减排。抓好列入“十一五”规划的重点减排项目，关闭不符合国家产业政策、高能耗、高污染的企业。支持涟钢、五江轻化等企业开展全国循环经济试点，鼓励企业推进产业循环、资源综合开发和能源综合利用。加强对县市区、重点企业的节能减排目标考核，坚决落实节能减排问责制。积极推进清洁生产，鼓励支持建筑节能，大力倡导机关节能。

切实强化环境治理。实施环境影响评价制度，强化“三同时”保证金制度，加大环境监控力度，完善环境应急机制。严格执行全省城镇污水处理设施建设三年行动计划，落实湘江流域水污染综合整治实施方案，加大资水、涟水、孙水和白马水库的综合治理力度，加快中心城区第二生活污水处理厂、垃圾填埋场二期工程、医疗废物处置中心、新化县城第二水源、各县市污水处理厂和垃圾填埋场建设。

加强自然生态保护。积极开展耕地开发整理，强化土地执法监察，确保耕地总量动态平衡，全面完成第二次土地调查和土地利用总体规划修编。继续整顿和规范矿产资源开发秩序，充分发挥市场在资源配置中的作用，提高资源利用效率。加快生态和城乡绿化建设，加强矿区植被保护和生态治理，严格征占用林地审批和林木采伐限额管理，全年完成造林9万亩，新增中心城区绿地65公顷，森林覆盖率达到42.08%以上。

（七）协调发展各项社会事业

加快推进科教兴市。深入开展科技创新年活动，大力推进创新型娄底建设，做好知识产权试点工作，争取我市列为省科技创新型试点城市。坚持教育强市，整合教育资源，调整教育布局，化解教育债务，关爱留守儿童，改善教师待遇，协调发展基础教育、职业教育、成人教育和高等教育，不断提升办学质量和水平。大力实施“人才强市”战略，营造尊重人才、关心人才的环境，加快人才引进、开发和培育，为科学发展、加速赶超提供强有力的智力支撑。

建立完善社会保障体系。千方百计扩大就业，积极做好高校毕业生、复转军人的就业创业工作，重点帮助零就业家庭和就业困难群体实现就业。完善医疗、养老等保障体系，不断提高城市低保补助标准，逐步实现农村低保的应保尽保。加大经济适用住房和廉租住房建设力度，切实解决低收入家庭和无房户的住房困难。加强以乡镇卫生院为重点的农村卫生服务网络建设，提高新型农村合作医疗受益水平，完善公共卫生服务功能，抓好社区卫生服务中心和服务站建设，规范医疗市场，构建和谐医患关系，逐步解决群众看病难、看病贵问题。

促进其他社会事业共同进步。扎实做好人口和计划生育工作，发挥计生协会作用，提高计划生育村居民自治合格率，加快全国人口文化新传播娄底示范基地建设，稳定低生育水平。继续推进广播电视村村通和有线电视数字化建设。切实做好第二次经济普查工作。深入开展全民国防教育和双拥共建活动，密切军民关系。发展老龄事业，重视残疾人工作，做好外事侨务、地方志、档案、民族宗教、新闻出版、对台、人防、红十字会、气象、地震等工作，重视和支持工青妇工作，大力发展妇女儿童事业。

（八）着力促进社会和谐稳定

高度重视安全生产。强化安全生产责任监管，落实安全生产“一票否决”，积极推进安全乡镇、安全县市、安全单位创建活动，强化对矿山、烟花爆竹、危险化学品、道路交通等重点领域的监管整治，坚决遏制重特大事故发生。加强饮用水源、食品、药品、农产品质量安全监管，切实保证人民群众生命健康与安全。

切实做好信访维稳工作。建立有效的信访工作长效机制，强化工作责任，规范信访秩序，加强源头控制，超前防范化解，坚决纠正损害群众利益的行为，切实维护好人民群众的合法权益，促进社会稳定。

加强社会治安综合治理。加强村民自治和社区居民自治建设，推进基层司法调解，健全社会治安防控体系，完善专项整治经常性工作机制，深入开展“打黑除恶”、打击“两抢一盗”、整治地下“六合彩”、禁毒扫黄等专项斗争。加强政府应急管理，加强群防群治，切实增强人民群众的安全感。

四、加强政府自身建设

推进科学发展，实现加速赶超，政府责无旁贷。当前，我们面临国际金融危机的严峻挑战，也拥有国家保增长、扩内需的重大机遇，挑战与机遇并存，困难与希望同在。我们必须坚定信心，肩负使命，努力建设人民满意政府，不断开创政府工作新局面。

深入解放思想，勇于开拓创新。继续解放思想，深入学习实践科学发展观。以科学发展观武装头脑，克服自满、畏难和狭隘思想，树立开放、进取和全局观念，努力增强驾驭市场经济、破解发展难题和做好群众工作的本领。发扬“坚韧、勤奋、尚德、自强”的娄底精神，用改革的思路、发展的办法解决制约发展的深层次矛盾和问题，使政府工作始终符合发展规律、时代要求和人民期待，始终充满生机和活力。

加快转变职能，致力优化服务。深入推进行政管理体制改革，坚持政企分开、政资分开、政府与中介组织分开，完善政府机构设置，理顺部门职责，切实转变政府职能。规范行政审批，推行政务公开，加快电子政务和政府信息化建设，逐步实现行政许可在线办理。加快财税改革，优化税收结构，推行阳光预算，增强财政保发展保民生的能力。精简文山会海，减少迎来送往，不搞形式主义，集中精力抓服务，一心一意促发展。落实《娄底市鼓励投资创业的若干规定》，全面实行刚性收费和全程代理服务，积极营造有利投资创业的发展环境，让一切有投资愿望的人找到投资机会，让一切有合作意向的人找到合作伙伴，让一切有创业能力的人找到创业平台。

坚持依法行政，做到执政为民。把遵守宪法和法律作为施政的根本原则。在市委领导下，认真落实市人大及其常委会各项决议决定，依法报告工作，主动与政协加强协商沟通，自觉接受人大法律监督、工作监督和政协的民主监督，提高议案、建议和提案的办理质量，广泛听取各民主党派、工商联、无党派人士和各人民团体的意见。严格按照《湖南省行政程序规定》和《娄底市重大行政决策程序规则》行使职权、履行职责。建立科学决策机制，完善群众建议征集制度、重大事项公示制度、听证制度和专家咨询制度，进一步提高依法行政水平。严格遵守和落实党风廉政建设责任制，进一步健全教育、制度、监督并重的惩治和预防腐败体系，以求真务实、民主公正、廉洁高效的形象取信于民。

切实转变作风，狠抓工作落实。继续开展创“人民满意机关”活动，推行政府工作绩效评估，建立科学的考核评价指标体系，完善工作量化考核办法。加强督查落实，强化责任考核，推行首问责任制、限时办结制、行政问责制，坚持用制度管权、管人、管事，以制度落实责任。加强公务员教育培训和管理，提高公务员素质，让勤奋学习、真抓实干、清正廉洁、厉行节约、高效服务在政府机关成为风尚，努力建设政治坚定、业务精湛、作风过硬、人民满意的公务员队伍。关心基层干部，支持基层工作，服务基层发展，最大限度地激发广大基层干部的积极性和创造性，最大限度地集中民智，听取民意，凝聚民心，共同推动富民强市的伟大事业。

各位代表！站在新的发展起点上，展望未来，发展蓝图令人鼓舞，赶超事业催人奋进。全市人民对政府工作的支持和期待，给了我们必胜的信心和无穷的力量，让我们在市委的正确领导下，抢抓机遇，乘势而上，开创科学发展的新局面，谱写加速赶超的新篇章！
